# Parent Perceptions of the Effects of Early Intensive Behavioral Interventions for Children with Autism

**DOI:** 10.3390/bs13010045

**Published:** 2023-01-04

**Authors:** Pamela Jean Giambona, Yi Ding, Su-Je Cho, Chun Zhang, Yangqian Shen

**Affiliations:** Graduate School of Education, Fordham University, 113 West 60th Street, LL 1008, New York, NY 10023, USA

**Keywords:** children with autism, early intensive behavioral intervention, applied behavioral analysis (ABA), parent perceptions, effects

## Abstract

The current study aimed to understand parents’ perceptions of the effects of early intensive behavioral intervention (EIBI) based on the principles of applied behavioral analysis (ABA) and the lasting outcomes for their children with Autism spectrum disorder (ASD). In particular, this study sought to examine parent perceptions of the relationship between the intensity of ABA interventions and current autism symptom severity, adaptive functioning, and school placement. The current study employed a convergent parallel mixed-methods design, which consisted of collecting, analyzing, interpreting, and combining both quantitative and qualitative data. Overall, results suggested that the intensity of previous ABA interventions was a unique predictor of current school placement. Additionally, results suggested that the intensity of previous ABA interventions was a unique predictor of adaptive skills, which was supported by parent interviews. However, the intensity of previous ABA interventions was not a unique predictor of current autism severity. Parent responses to interview questions revealed the imperative nature of the interventions and their effect on service delivery for their children with ASD. Overall, this study provided an increased understanding of parents’ perceptions of the effectiveness of EIBI, which in turn may be central to understanding service utilization.

## 1. Introduction

Individuals with autism have impairments in many areas of development that can lead to social, communication, and behavioral challenges. Currently, there are numerous treatments available to address the symptoms of autism; however, research has demonstrated that early intensive behavior intervention (EIBI), which is based on the principles of applied behavioral analysis (ABA), may be the most effective [1,2]. The timing, intensity, and type of early intervention services received may be associated with overall immediate outcomes related to gains in IQ, adaptive behaviors, and school placement [3,4,5]. 

### 1.1. Autism Spectrum Disorder

Autism spectrum disorder (ASD), also commonly referred to as autism, is a complex neurodevelopmental disorder that is characterized by impairments in social communication and interactions and by the presence of restricted and repetitive patterns of behavior, activities, or interests [6]. ASD is characterized by some core symptoms related to language inability, behavioral deficits, sensory symptoms, and emotional and cognitive challenges. Autism symptom severity refers to the intensity of core symptoms of ASD. According to the Diagnostic and Statistical Manual of Mental Disorders, Fifth Edition (DSM-5) [6], autism is a single diagnosis with three levels of severity that are intended to illustrate an individual’s abilities as well as their support needs based on the level of social communication and interaction impairments and repetitive, restricted behaviors, activities, or interests [6]. 

Within the United States, autism remains the fastest growing childhood neurodevelopmental disorder, with prevalence rates more than doubling in the last two decades [7,8,9]. In 2012, one in 69 children at age 8 years was diagnosed with autism [10], and in 2014 the prevalence was one in 59 children at age 8 [11,12]. The disorder disproportionately affects males, who are four times more likely to be diagnosed with autism than females [10,13,14]. Consistently, the global prevalence of autism has ranged between 1% and 2% [15,16]. According to Matson and Kozlowski [17], the dramatic increase in ASD diagnoses may be attributed to expanded diagnostic criteria leading to a broader population being diagnosed than the previous population, better detection (e.g., parents are more aware of early signs of ASD and request diagnostic evaluations for their children much earlier in the trajectory), and less stigma associated with the disorder (e.g., the public is more open to individuals with ASD).

### 1.2. Adaptive Behaviors

Adaptive behaviors are skills that individuals need to function independently in their environment [18,19]. They represent the ability to translate cognitive potential into real-world skills [20] and are crucial to adjustment and normal functioning; thus, they are daily living skills that promote independence, social acceptability, and quality of life [21]. Research has shown that individuals with ASD tend to display deficits with adaptive functioning [18,22], as their adaptive behaviors are likely impaired or delayed [23,24]. Matson and Shoemaker [22] found that as the severity of ASD symptoms increases, the level of adaptive skills decreases.

To be specific, individuals with ASD have significant difficulties in adaptability and transitions in their daily routines [25,26]. Iadarola et al. [26] suggested that difficulties with adaptability and transitions can result in challenging behaviors such as noncompliance, tantrums, aggression, and self-injury. Problems with adaptability may stem largely from autism-related rigidity; as Whitman and Ekas [27] observed, when rigidity is high, it can be expected that adaptability will be low. Additionally, the adaptive skill of communication often presents as a deficit for individuals with ASD [28], and language delays are common when compared to their typically developing peers [29,30]. These social communication deficits may be attributed to their difficulty understanding and using cultural norms as well as interpreting gestures, body language, and facial expressions [31]. Moreover, Pugliese et al. [20] indicated that individuals with ASD have the lowest adaptive behaviors in socialization skills [28], which may be attributed to their atypical attentional engagement to social stimuli [32], lack of attention to social contexts [32], and deficits in processing social-emotional information [33].

### 1.3. School Placements

School placement, also commonly referred to as educational placement, is the classroom setting in which a child receives educational services. According to the Individuals with Disabilities Education Improvement Act [34], students must be educated within the least restrictive environment. The principle of educating students in their least restrictive environment is universally accepted; however, there are concerns with how the principle is interpreted. Kurth [35] noted that when students have less access to typical peers, general education curriculum, and activities, the setting is considered more restrictive.

In current education system, children with autism are placed in different types of classrooms. Approximately 7.4% of children with autism in the United States are placed in specialized schools that serve only students with autism and related disabilities [35,36,37]. These schools often have a low teacher-student ratio with a highly individualized curriculum. Students with autism also may be placed in self-contained classrooms, which is led by a teacher with specific skills in educating students with various needs. Approximately 34.8% of students with autism are educated in self-contained classrooms [35]. It is generally understood that self-contained classroom teachers are better able to tailor the educational experiences to individual students’ needs, but it might not be appropriate to meet the needs of all children with ASD, as their needs are too diverse to tailor the curriculum in this setting [38]. Inclusion classrooms are another setting in which students with autism may be placed. An inclusion classroom is a classroom with both a general and a special education teacher and a specific ratio of children with and without disabilities. Additionally, approximately 20.4% of students with autism are educated in general education settings, where education teachers typically do not have special education training [35,38]. Overall, research on which setting is most appropriate for students with ASD has been inconclusive. Depending on the individual student’s abilities, each environment can be considered either the most or the least restrictive environment. Thus, the least restrictive environment can vary depending on the severity of symptoms of ASD students [39]) and it is imperative to consider each student’s individual abilities and skills when considering school placements. In this paper, we consider that less restrictive school placement indicates that the learners with ASD need less intensive support.

### 1.4. Early Intensive Behavioral Interventions

ASD is a lifelong disorder that has no known cure, but there are available treatments to help address the associated symptoms [40]. Two of the current treatments available for individuals with ASD are pharmacological and behavioral therapy [15,41]. Medication has been found to provide some relief for behavioral symptoms, such as tantrums, self-injurious behaviors, aggression, hyperactivity, and repetitive behaviors [41,42]. However, it is not the primary treatment modality for ASD as research has suggested the lack of substantial benefits [15,43]. Reichow et al. [15] claimed that therapy based on developmentally informed behavioral treatments is the most beneficial for individuals with ASD [41]. Additionally, they suggested that developments in behavioral treatments have outpaced medical or pharmaceutical advances. One of the primary therapy treatment options is EIBI, an educational service offered to children from birth up to age 6 years [44] based on the principles of ABA [1,15].

ABA interventions are generally understood as intensive programs that require approximately 30 h per week of instruction for at least 2 years. They are individualized and comprehensive, and address all skill domains [4]. When applying ABA to autism intervention, the original principles of learning were used to target the core behavioral deficits in autism [45], and to facilitate gains in social, emotional, intellectual, and adaptive functioning [1,5,15,46,47,48].

ABA therapy can lead to noticeable and lasting functional improvements by using principles that foster developmentally important skills and minimize challenging behaviors [3,49]. To receive the most notable effects of ABA therapy, the procedures need to be strictly administered [3]. The most substantial effects can be achieved when treatment begins before age 4 years [50]. Treatment needs to be intensive, with the weekly sessions totaling 20 to 40 h per week for at least 1 to 4 years [15,51,52] and with parents involved in treatment, who frequently serve as co-therapists and supply additional lessons and treatments at home.

### 1.5. Effectiveness of Intensive Early Behavioral Interventions

Studies have demonstrated that ABA can have significant effects on IQ gains, adaptive functioning, and later school placement [5]. Experimental research has been conducted in school, home, and community environments with therapy being provided by both therapists and parents [1,5,53,54,55,56].

A meta-analysis was conducted to examine the immediate effectiveness of ABA interventions in young children with autism [57]. The study looked at 11 controlled studies (*n* = 344 participants) with a pre-/post-test design for evaluating ABA therapy with children ages 10 years or younger of which more than half were male. On average, children in the ABA groups received intensive intervention from 12.5 to 38.5 h per week over a 10-month to 2-year period. The control groups received a less intensive version of ABA therapy. The results showed statistically significant differences between the experimental and the control groups for full scale IQ and adaptive behaviors. The results of this meta-analysis revealed that the experimental groups outperformed the control groups on measures of IQ and adaptive skills, suggesting support for the effectiveness of intensive ABA intervention.

Researchers have found that children who previously received ABA therapy were more likely to be placed in less restrictive settings and required fewer school supports [1,48,53,55,56]. For example, Cohen et al. [53] examined if receiving ABA therapy was related to school placement with a sample of 42 children comprising an experimental group (*n* = 21) and a control group (*n* = 21). The experimental group received 35 to 40 h per week of the behavioral intervention for 3 or more years, and the control group did not receive the behavioral intervention. Results from the study suggested that the children who received the ABA intervention were more likely to be placed in a general education setting when compared to the control group. Specifically, 17 of the 21 children who received ABA intervention and 1 of 21 in the control group were included in general education classroom settings. Further, 29% were included in regular education classrooms without supportive services [53].

Given the demonstration for the immediate effectiveness of EIBIs, researchers have begun to study the long-term effectiveness of EIBIs. However, there is limited research on the long-lasting effects of ABA interventions due to the few follow-up studies conducted. One of the first follow-up studies assessed the long-lasting effects was conducted by McEachin et al. [2]. Specifically, Lovaas [1] conducted initial research to examine the effectiveness of ABA therapy for 38 children with autism by comparing an experimental group (*n* = 19) and a control group (*n* = 19). McEachin et al. [2]) reassessed the same participants from Lovaas’s study after their treatment were discontinued for a minimum of 4 years. At the point of reassessment, children in ABA-group maintained gains, as they demonstrated higher IQ scores and adaptive behavior scores than the control group. Many of the mainstreamed participants from the first study continued to succeed in their placements. Grindle et al. [58] also conducted a follow-up study of participants who had previously received ABA therapy. The sample consisted of 29 participants, with 11 in the experimental group and 18 in the control group. IQ scores and adaptive skills were reassessed 2 years after the termination of treatment. Results from this study suggested that when compared to the control group, the experimental group had statistically significant larger effects for adaptive skills [58].

These studies demonstrated that when ABA therapy was delivered at an early age, it was more likely to produce significant improvements. Likewise, Howard et al. [59] found that when participants received ABA interventions, they were two times as likely to score in the average range on IQ, language, and adaptive behaviors scales. Kovshoff et al. [60] reported that participants who were mainstreamed after initial treatment continued to be integrated at follow-up. These follow-up studies support the findings that early intensive ABA interventions benefit individuals with ASD. The EIBIs provided immediate results and maintained results for short periods of time, with follow-up studies examining the long-term effectiveness typically reassessing individuals after 1 to 2 years.

### 1.6. Parent Perception

Parents’ engagement and cooperation are determinant to the effectiveness of EIBI services for children with autism, because they generally take an important role of providing direct interventions to promote their children’s generalization of learning at home [61,62]. Parents are also involved in the collaboration with various services providers and organizations for intervention planning and supervisions [61,63,64]. Taking parents’ perceptions into consideration to evaluate the effectiveness of service is critical. Solish and Perry [65] indicated that both similarities and discrepancies were recorded between parent and service provider report regarding the effectiveness of EIBI services. The discrepancies may be related to various challenges, such as the difficulty in establishing effective collaborative partnerships, the inconsistent expectations about service outcome, the cultural considerations in the EIBI service procedure, and the parents’ levels of self-efficacy [66,67]. Those challenges may impact the family’s adjustment in implementing interventions at home. Thus, a better understanding about parents’ perceptions could facilitate providers ground their services based on the realities and then make accommodations on intervention to optimize the effectiveness of EIBI services [64].

### 1.7. Statement of Problem

Research has revealed that knowledge of parents’ perceptions regarding the effectiveness of EIBI services is key to understanding service utilization and its corresponding outcomes [49,68]. However, there has been limited research on parent perceptions of the lasting outcomes of previous ABA intervention. Thus, the focus of this study was on parents’ retrospective perceptions of past EIBI services and current outcomes for their children with ASD. Parents of children with ASD provided information about their child’s current autism symptom severity, adaptive functioning, and school placement as well as the intensity of the past received EIBI. It was expected that a deeper understanding of parents’ retrospective perceptions of the effectiveness of EIBI would be central to understanding service utilization and its related lasting outcomes.

Three research questions were explored in the present study: (1) Did the intensity of previous ABA interventions, as reported by parents, explain the variance in manifestations of current autism severity? (2) What was the relationship between current adaptive skill levels and the intensity of previous ABA interventions? (3) What was the relationship between current school placements and the intensity of previous ABA interventions?

Based on a review of the literature on the effects of ABA-based early interventions on autism severity, adaptive skills, and school placement of children with ASD, the following hypotheses were developed: (1) the intensity of previous ABA interventions would uniquely explain variance in the manifestation of autism severity. Further, a higher intensity of ABA interventions would be associated with lower autism severity levels whereas a lower intensity of ABA interventions would be associated with higher autism severity levels; (2) the intensity of previous ABA interventions would be associated with current adaptive skill levels as rated by parents; and (3) the intensity of previous ABA interventions would be associated with current school placement as rated by parents.

## 2. Method

**Figure 1:**
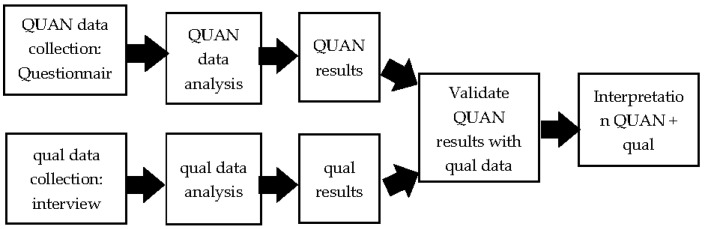
Procedural Diagram of the Proposed Convergent Parallel Design.

This study used a convergent parallel design [69]. Within this design, both quantitative and qualitative data were collected simultaneously (QUANT + qual) and were merged for an overall interpretation (See Figure 1). This design was used in order to gain a deeper understanding of parent perceptions of the effectiveness of EIBIs for children with autism, as this method helped to provide a more comprehensive view as well as corroborate study findings. Collecting qualitative data helped to identify emergent themes as well as to validate and expand on quantitative results with corroborative evidence from different methods [70,71].

### 2.1. Participants

The current study recruited parents of children diagnosed with ASD based on either DSM-IV-TR or DSM-5 criteria. The participants’ children ranged in age from 6 through 18 years, and data regarding gender, ethnicity, and socioeconomic status were collected. All participating children were born in the United States and were raised in the state of New York.

A power analysis was conducted based on three control variables (child’s current age, gender, and diagnostic severity), two independent variables (length of received early intervention and hours of ABA treatment), and three outcome variables (Autism Index, General Adaptive Composite, and School Placement) with a power of 0.8, and an alpha of 0.05 to determine the sample size for quantitative data. Approximately 91 participants should be included [72], while the number of participants in this study was 72, which were all used in the regression analyses. Regarding qualitative data, the guidelines of Onwuegbuzie and Collins [73] were employed for interviews. An adequate sample size for interviews was 12 participants ([74], but 29 parents participated in the interviews.

All participants were parents of children with ASD. A majority of the participants were Caucasian, were married, had some level of college education, and were employed for wages. The participants were recruited through the researchers’ professional and personal network; thus, the participants were predominantly individuals with some college education or higher. The sample might not truly represent the demographics of the US population. Child characteristics were also defined in the sample. Most of the children were males, were between the ages of 6 and 8 years, and had been diagnosed with mild ASD between the ages of 3 and 5 years. More specific demographic information is presented in Table 1. A breakdown of school placement based on diagnostic severity level is presented in Table 2.

### 2.2. Measures

#### 2.2.1. Demographic Variables

The demographic survey was derived from the Hume et al. [44] questionnaire with the consent of usage. Parents reported demographic information regarding ethnicity, marital status, occupations, and education levels. They also answered questions about their child’s age, gender, age of diagnosis, diagnostic severity level, and EIBI history. EIBI history included the age at which services began, the location where services occurred, and the length of time and hours per week the child received intervention. The EIBI history was recorded to determine the intensity of the interventions. Lastly, parents were asked to rate the effectiveness of EIBI for their child.

#### 2.2.2. Parent Interview

The researcher developed the semi-structured parent interview questions. Questions were based on elements regarding parent perceptions of early intervention. Semi-structured interviews were performed at the convenience of the participant to further understand (a) behaviors before ABA interventions were received as well as current behaviors; and (b) parents’ perceptions of the ABA therapy their child received during early intervention (*n* = 29). After the interviews, parent responses were summarized.

#### 2.2.3. Autism Symptom Severity

The Gilliam Autism Rating Scale–Third Edition (GARS-3) [75] was used to assess the severity level of ASD. The GARS-3 is one of the most widely used assessments of ASD, as it can be used to both identify autism and assess its severity in individuals between the ages of 3 and 22 years. The GARS-3 contains 56 items describing the characteristic behaviors of individuals with autism. The items are rated on a 4-point Likert scale ranging from 0 (not at all like the individual) to 3 (very much like the individual). In this study, parent perceptions of autism severity were collected by administering the GARS-3 rating scale to parents. The Autism Index (AI), a total score, is derived from the ratings provided on the likelihood of the individual having an ASD diagnosis. The AI was interpreted through standard scores with a mean of 100 and standard deviation of 15. The larger the standard score, the more severe the diagnostic level. For the AI scale, scores greater than 100 fall in the DSM-5 severity Level 3 range, scores from 71 to 100 in the Level 2 range, scores from 55 to 70 in the Level 1 range, and scores less than or equal to 54 are not associated with ASD. Test–retest reliability coefficients of the GARS-3 exceeded 0.90 for the AI, demonstrating good reliability. The GARS-3 also demonstrated good internal consistency with coefficient alphas on the AI exceeding 0.93. Binary classification studies indicated that the GARS-3 enables accurate discrimination of children with ASD from children without ASD (sensitivity = 0.97, specificity = 0.97).

#### 2.2.4. Adaptive Behaviors

The Adaptive Behavior Assessment System–Third Edition (ABAS-3) [76] was used to assess adaptive behaviors and related skills for people across the life span from birth to 89 years 11 months old. The parent form was used in this study. The items are rated on a 4-point Likert scale ranging from 0 (is not able) to 3 (always). In this study, parent perceptions of adaptive skill outcomes were collected by administering the ABAS-3 rating scale to parents. The total score, referred as general adaptive composite (GAC), is derived from the ratings and is interpreted by standard scores. The higher the standard score, the higher the level of the child’s adaptive behaviors. Scores greater than or equal to 120 were in the high range, from 110–119 in the above average range, 90–109 in the average range, 80–89 in the below average range, 71–79 in the low range, and scores less than or equal to 70 in the extremely low range. The ABAS-3 has been widely used and accepted as a reliable and valid measure of adaptive behaviors. The test–retest correlation is 0.84 for GAC. Additionally, the ABAS-3 demonstrated good internal consistency with coefficient alphas ranging from 0.91 to 0.99.

#### 2.2.5. School Placement

Information on school placement and levels of support in the classroom was collected via parental reports. For school placement, a lower score was associated with a less restrictive setting while a higher score was related to a more restrictive setting (1 = public school, 2 = special needs school, 3 = home program, 4 = residential program, 5 = medical/therapeutic facility). When a child has an autism diagnosis, the child typically requires support within the classroom. The amount of support required can vary depending on the student’s cognitive, adaptive, behavioral, and emotional skills.

### 2.3. Procedures

Following approval of the study by Fordham University’s Institutional Review Board (IRB), participants were recruited through Amazon Mechanical Turk, personal contacts, local agencies, and school districts serving individuals with an ASD diagnosis and their families. Personal contacts were approached directly. To preserve confidentiality and anonymity of families, school districts distributed the Fordham University IRB approved recruitment letter through an email blast, and anonymous local agencies were provided with questionnaire packets to be distributed to families. Each packet contained the questionnaires as well as stamped, self-addressed envelopes to send materials back to the researcher at their convenience. The participation of this study is voluntary.

A subset of the complete sample (*n* = 29) participated in the interview portion of this study. Interviews were conducted at the convenience of the participants (e.g., in-person, telephone, or written). For the interview, the participants completed the demographic questionnaire and interview questions. Given the simple, quick, and inexpensive nature of field notes, the researcher took notes and summarized participants’ responses. According to Ashmore and Reed [77], field notes are a good option when used with other methods in a mixed-methods design. Participants who participated in the interview portion also completed written consent forms, the GARS-3, and ABAS-3 via U.S. mail.

After all data were collected, interview and questionnaire data were entered into an SPSS database. Each participant was assigned an ID number, and collected data were stored in a locked filing cabinet to maintain confidentiality. The participant’s signature on the IRB forms served as consent to include their responses in this study.

## 3. Results

Quantitative and qualitative data were analyzed separately (QUANT + qual), and the results were merged for overall interpretation. A side-by-side comparison was employed and present in the results section. Quantitatively, demographics, correlations, and regression analyses were conducted for the 72 participants. Demographics and exploratory correlation analyses were conducted to examine the relationship between all variables. Hierarchical regression analyses were used to test all three hypotheses. Qualitatively, the researcher summarized participant responses. The responses were then examined and sorted based on themes.

### 3.1. Overview of Data

The total number of participants recruited for this study was 127. Of the total recruited, 52 recruited parents were eliminated because they did not meet the criteria for the study, as their child was not within the study’s age range and/or did not receive ABA therapy during early intervention. Three recruited parents were further eliminated due to failing to complete the questionnaires, resulting in a final sample size of 72 participants. Of the 72 final participants, mean scores were entered for missing items on the questionnaires. Visual inspection of the data for the 72 participants revealed a normally distributed data set. Of the 72 participants, 29 participants completed the interview portion of the study.

### 3.2. Descriptive Analyses of Variables

Descriptive data for all outcome and predictor variables are presented in Table 3. Descriptive statistics indicated that children in this study averagely demonstrated a DSM-5 severity Level 2, suggesting that they required substantial support (*M =* 86.4, *SD* = 18.9), and demonstrated below average adaptive skills (*M =* 83.5, *SD* = 22.5). Additionally, preliminary descriptive statistics indicated that children’s school placement was less restrictive (*M =* 1.7, *SD* = 1.3). Further examination of school placement showed that 49 parents reported that their child was currently placed in a public-school setting (68.1%), 10 children were in special needs schools (13.9%), four were in home programs (5.6%), three were in residential facilities (4.2%), and six were in medical/therapeutic facilities (8.3%). Based on the descriptive statistics, children in this study received moderate intensity ABA intervention (*M =* 12.5, *SD* = 9.5). Specifically, 26 children received low intensity intervention (36.1%), 27 received moderate intensity intervention (37.5%), and 19 received high intensity intervention (26.4%). Further, the results indicated that 49 children received less than 2 years of intervention (68%) and 23 children received from 2 to 4 years of intervention (32%).

### 3.3. Exploratory Correlations

Pearson correlation analyses were used to examine the relationships between early intervention variables (See Table 4). The selected results with moderate or strong correlations were presented as follows. First, there was a moderate, positive correlation between the age at diagnosis and the age at which EIBI began, *r* = 0.55, *p* = 0.000, suggesting that early diagnosis led to early intervention. Second, there was a moderate, negative correlation between the age of EIBI and the length of time in months that intervention was received, *r* = −0.45, *p* = 0.000, suggesting that the younger a child at the beginning of EIBI, the greater the number of months of intervention. Additionally, children’s current adaptive skill level was found to be moderately, negatively correlated with diagnostic severity, *r* = −0.42, *p* = 0.000, suggesting that children with higher diagnostic severity levels had lower adaptive skill levels. Additionally, there was a moderate, positive correlation between parents’ perception of the effectiveness of previously received EIBI and their report of the child’s current overall quality of life, *r* = 0.49, *p* = 0.000. This suggests that parents who perceived ABA intervention to be effective also perceived their child as having an increased quality of life.

### 3.4. Regression Analyses

For each model, collinearity diagnostics were performed as part of each regression through the assessment of the variance inflation factor (VIF), tolerance, and condition index. All models met the guidelines and therefore multicollinearity was determined not to be present. Additionally, the models were evaluated for the presence of univariate outliers by visual inspection of scatterplots and screening for z-scores +/− 3.04, and no univariate outliers was found in this study. There were no multivariate outliers for any of the models, as the Mahalanobis distance did not exceed the critical value of 20.52 (*p* < 0.001) [78]. Additionally, visual inspection revealed that the models were consistent with the assumptions of normality, linearity, and homoscedasticity.

To explore whether the intensity of previous ABA interventions, as reported by parents, explain the variance in the manifestation of current autism severity, a hierarchical regression was conducted examining the relationship between hours of EIBI, length of time of EIBI, and current autism symptom severity (See Table 5). The control variables of age, gender, and diagnostic severity were entered into the model first to account for any shared variability between the control and the predictor variables. The first stage of the model was statistically significant, suggesting that the control variables accounted for a total of 11.5% of the variance in current autism symptom severity, *R*^2^ = 0.115, adjusted *R*^2^ = 0.076, *F*(3,68) = 2.952, *p* = 0.039. To determine the impact of the intensity of EIBI on current autism symptom severity, hours per week and length of time in months of EIBI were added to the second model. This did not result in a statistically significant increase in the explained variance, *R*^2^ = 0.127, adjusted *R*^2^ = 0.061, *F*(5,66) = 1.916, *p* = 0.103. The result suggests that the hours per week and length of time of EIBI did not uniquely contribute to current autism symptom severity. Thus, the hypothesis that the intensity of previous ABA interventions predicts the variance of current autism severity was rejected.

To examine the relationship between adaptive skill levels and the intensity of previous ABA interventions, a hierarchical regression was conducted through two model stages (See Table 6). The first stage of the model was statistically significant, suggesting that the control variables of age, gender, and diagnostic severity accounted for a total of 28.7% of the variance in current adaptive functioning, *R*^2^ = 0.287, adjusted *R*^2^ = 0.255, *F*(3,68) = 9.117, *p* = 0.000. The child’s current age (β = −0.363, *p* < 0.01) and the child’s diagnostic severity (β = −0.253, *p* < 0.05) displayed significant negative relationships to adaptive functioning, and both of them remained significant throughout the entire model. The second stage of the model was also statistically significant, suggesting that the intensity of ABA intervention accounted for an additional 14.3% of the variance in current adaptive functioning, *R*^2^ = 0.430, adjusted *R*^2^ = 0.386, *F*(5,66) = 9.942, *p* = 0.000. The length of time in months a child received EIBI was statistically significant (β = −0.384, *p* < 0.01), indicating that the length of intervention did add a unique contribution to current adaptive functioning. Taken together, the results supported the hypothesis that the intensity of previous ABA interventions was associated with the child’s current adaptive skill level. Specifically, the longer the previous intervention lasted, the higher the current adaptive functioning.

A hierarchical regression was conducted to examine the relationship of the intensity of the ABA intervention and current school placement (See Table 7). Similarly, two different stages of the model were examined. The first stage of the model was not statistically significant, suggesting that the control variables did not account for variance in current school placement. After adding the hours per week and length of time in months of intervention to the second model stage, the model was statistically significant, suggesting the intensity of ABA intervention accounted for an additional 12.3% of the variance in current school placement, *R*^2^ = 0.159, adjusted *R*^2^ = 0.096, *F*(5,66) = 2.502, *p* = 0.039. The length of time in months a child received EIBI was statistically significant, (β = −0.303, *p* < 0.05). Thus, the results showed that the length of time a child received ABA intervention uniquely contributed to their current school placement.

### 3.5. Qualitative Analyses

The primary objective of this qualitative portion of the mixed-method analysis was to expand on quantitative results as well as investigate parent perceptions of EIBI. Based on field notes, two broad themes emerged: Nature of the Intervention and Adaptive Skills.

#### 3.5.1. Nature of the Intervention

The nature of the intervention was defined as the logistical aspects of the ABA intervention, such as timing, consistency, and collaboration.

Timing: Many parents indicated that the age at which intervention began was extremely important. Parents indicated that they felt the earlier the intervention began, the better the results for their child. For example, one parent stated, “The earlier you start, the better chances of seeing progress and changes in behavior.” Additionally, one parent felt it was a wonderful tool to use before beginning school.

Consistency: Numerous parents discussed how the principles of ABA and its requirement for consistency was fundamental to their child’s success. For example, one parent stated, “ABA has provided the framework and structure that enables my son to learn and acquire new skills while feeling more confident through reinforcements.” Four parents commented on how vital consistency was for the success of their child, and six parents indicated the need for multiple types of consistency. Specifically, one parent pointed out the need for consistency between all team members working with the child. Another parent commented on the need for consistency between the home and the school. These ideas are imperative, as they emphasize the need for consistency in the interventions as well as consistency in the way different individuals work with the child.

Collaboration: throughout the interviews, it became apparent that parents felt that collaboration between all individuals interacting with their child and between parents and providers was critical. Two parents commented that the collaboration required for EIBI helped to educate parents that everyone’s behaviors can contribute to the child’s behaviors.

Three parents expressed negative perceptions regarding the ABA interventions their child received. In particular, two parents reported that the tools used in the ABA intervention could be frustrating for both children and parents. One parent indicated that the nature of the intervention was too repetitive and at times reinforced negative behaviors. Parents also commented on the difficulty of working with and finding providers to conduct sessions. Specifically, one parent indicated that having multiple therapists providing treatment in the home led to a “revolving door.” Due to their difficulties with aspects of the ABA interventions, these parents did not feel that EIBI provided lasting effects or improvements in the quality of life of their child.

#### 3.5.2. Adaptive Skills

In six interviews, the theme of adaptive skills became apparent. Parents discussed how vital EIBI was for the development and growth of their child’s adaptive skills. In particular, children’s communication and social skills were aided by the interventions. Four parents discussed how ABA intervention contributed to their child’s adaptive skill of communication. One parent stated, “He is able to express himself a bit better, his wants and needs are able to be expressed, general communication and cognitive abilities improved.” Additionally, one parent expressed that daily living skills in general were helped by EIBI. The parent indicated that all aspects of life were eased, such as going to doctors and parks with her child, which she attributed to the work carried out during EIBI. Further, two parents discussed how ABA intervention contributed to the growth of their child’s social abilities. Parents’ statements suggested that they found EIBIs to be a beneficial treatment for adaptive skills.

## 4. Discussion

### 4.1. Lasting Effects of ABA Interventions

Results from this study showed that the intensity of previous EIBI was not a unique predictor of current autism symptom severity, which is inconsistent with the literature, as research shows that children who received intensive early ABA intervention demonstrated higher skill domains, more positive behaviors, and growth in daily living skills, social behaviors, and language skills [3,5,53,54,55,57,79]. One possible explanation for this inconsistency may be related to the sample. In this study, a heterogeneous sample was used in which there was a wide range of diagnostic severity levels, which was not the case in many of the controlled studies in the literature. Additionally, the intensity of ABA interventions in this study was significantly lower than the intensity levels reported in the current literature. In this study, children received an average of 12.5 h per week of EIBI, which is considered a moderate level of intensity. However, this level may not have been intense enough to produce lasting outcomes in terms of symptomology. Additionally, some autism symptoms can present as covert behavior, which can make it more challenging for parents to judge if there is a change in symptomology that cannot be directly observed.

Based on the results of this study, the length of time EIBI was received is associated with current adaptive skill levels, which is consistent with findings in the literature [58,80]. It is likely that these results are consistent due to the number of months children received EIBI. Parents reported that children received an average of 15.8 months of EIBI, which is consider intense intervention. Additionally, children can learn adaptive skills through EIBI, which can lead ABA intervention to be a unique predictor. Given that adaptive skills are overt behaviors that parents can observe, they are able to see their child’s growth in these areas. Additionally, the relationship between the intensity of ABA interventions and school placement was demonstrated in this study, particularly revealing the importance of the length of time of ABA intervention. This is consistent with findings in the current literature that a child who receives intense ABA intervention may be placed in a less restrictive school setting and may require minimal school supports [1,48,53,55,56,60,81]. These results are likely consistent with the literature due to the number of months children received EIBI, which provides the child with more support and leads to better outcomes. It is also possible that ABA intervention teaches children from an early age the skills required in classroom settings.

### 4.2. Nature of the Intervention and Adaptive Skill

Regarding the nature of the intervention, parents described their experiences with EIBI and its service delivery. Parents discussed the importance of timing, which is consistent with the current literature [4,50]. In particular, parents described that the earlier an intervention was started, the greater the success they observed for their child. Children are able to benefit the most from services that are provided earlier in life, and parents are able to see the rapid change in their child’s ability levels. Parents also described the importance of consistency and collaboration in service delivery, which is consistent with the current literature [4,15]. ABA interventions are generally provided under strict guidelines, and therefore parents are likely to report similar experiences with the interventions.

The theme of adaptive skills emerged during multiple parent interviews. Specifically, parents reported that ABA interventions provided support to the development and growth of their child’s adaptive skills. Parents reported that life skills and social skills were taught throughout the course of EIBI. Additionally, parents reported that previous EIBI was beneficial to the child’s current adaptive skill level. This qualitative outcome is consistent with findings in the literature that ABA intervention has been found to contribute to growth in adaptive skills [5,58,80]. This is likely the case because parents are able to directly observe and judge their child’s growth in adaptive skills.

### 4.3. Comparison and Integration of Findings

Overall, the results of this study showed mixed results. A side-by-side comparison of quantitative and qualitative outcomes related to predictor variables is presented in Table 8. In addition, there were connections between exploratory findings of this study and the emerging qualitative themes. Regarding the theme of the nature of the intervention, multiple parents stressed the importance of consistency, collaboration, and the timing of the intervention. Specifically, the timing of the intervention was consistent with the exploratory correlations. There was a weak negative correlation between the age that EIBI began and parents’ perceptions of its effectiveness. In this study, parents of children who began EIBI at a younger age perceived the intervention to be more effective.

## 5. Limitations and Future Research

The foremost limitation of this study was the sample. First, a power analysis was conducted to determine an adequate sample size of approximately 91 participants, but the number of participants included in the final hierarchical regression analyses was 72. However, due to the mixed methods nature of this study, the 29 interviews helped to build upon the richness of results. Second, this research utilized a convenience sample, which may limit the study’s findings because the participants voluntarily chose to participate. Those who elected to participate might have had an intrinsic interest in the study, might have participated in previous studies, or were enticed by the compensation for participating. This could have resulted in self-selection bias. The researcher attempted to lessen the possibility that participants elected to participate because of compensation by keeping it minimal. Third, the demographics of this sample should also be considered. To qualify for this study, participants had to reside within the state of New York. This was decided due to the variability of available interventions and service implementation across different states. Requiring that all participants reside in New York decreased the generalizability of the results. The participants were parents of children and adolescents with ASD from a very wide age range. It might pose a potential issue because learners with ASD might encounter very different challenges at different ages. Forth, the parents in the interview sample were all mothers. This was a disadvantage because mothers are not the only caregivers of children. Given the limitations in study sample, future research could take this study further by altering the sample with lager sample size, less broad demographics, a smaller age range, and involving all caregivers. Fifth, we classified the school placements into different categories (e.g., a less restrictive setting to a more restrictive setting). This is only an approximate approach to indicate the level of support that a student might need. It is important to note that school placement might reflect the parents’ own preferences. Sixth, the data were collected from our parents’ retrospective report on their perspectives about the effects of early intensive behavioral intervention that their children received. The data did not reflect immediate follow-up on the children who received EIBIs. In addition, the participants’ children might receive EIBIs at different points of time, which is not controlled by the present study.

Another major limitation of this study was the method of data collection. This study utilized self-report through both interviews and questionnaires, thus, participants could have responded in a socially desirable way or in a manner that they believed would be viewed as favorable by the researcher. Additionally, participants completed the questionnaires independently and did not have the opportunity to ask questions on demand. In an attempt to mediate this limitation, participants were provided with both the researcher’s and the mentor’s contact information, but it could not eliminate this limitation. Thus, researchers could use alternative forms of data collection (e.g., direct outcome measures of children and adolescents with ASD) to verify the results in this study.

Additionally, the choice of independent and control variables could be a limitation of this study. In terms of independent variables, hours per week and length of time in months were chosen to describe the intensity of the interventions received. However, other variables could have been used to determine the intensity of EIBIs. For example, the settings in which EIBI was received could have been used to help determine the intensity in order to understand the relationship between previous services and lasting outcomes. Thus, future research could examine the effects of other variables that could be used to determine the intensity of EIBIs.

Researchers may also want to examine the multicultural aspects of service utilization. Specifically, researchers could examine if there are any cultural differences in parent perceptions of ABA interventions.

## 6. Conclusions and Implications

This study adds to the literature on parent perceptions of the effectiveness of EIBIs through the use of a mixed-methods design. Specifically, this study adds to the growing body of knowledge related to the effects of EIBIs on children’s autism symptom severity, adaptive functioning, and school placement. Although this study did not show a significant relationship between EIBIs and current autism symptom severity, it did find a relationship with current adaptive skill level and school placement. Most importantly, this study was able to gain a clearer understanding of parents’ perceptions of these interventions through the interview process. Parents expressed that EIBIs were most effective for their child when provided at an early age. Additionally, parents believed that EIBI was most beneficial when there was a consistent and collaborative nature to the intervention among home, school, and all team members. The interviews revealed that parents described EIBIs as having the greatest effect on adaptive behaviors, which was consistent with the statistical results.

This study has additional implications for researchers. It is critical for researchers to determine why EIBIs have potentially long-lasting effects on adaptive functioning. Within the last several decades, the prevalence of ASD has increased dramatically, and one of the core symptoms of ASD is decreased adaptive functioning. If researchers can determine what intensity of EIBIs is needed to produce positive gains in adaptive functioning for children with ASD, this will contribute to providers and caregivers successfully meeting these challenges.

## Figures and Tables

**Table 1:** Demographic Characteristics of Participants.

Variable	*n*	*%*
Ethnic Identification		
African American	3	4.2
Asian American	6	8.3
Caucasian	37	51.4
Hispanic	20	27.8
Other	6	8.3
Parent Highest Education		
Never Attended	0	0.0
Elementary	3	4.2
Some High School	3	4.2
High School Graduate	2	2.8
Some College or Technical School	10	13.9
College Graduate	36	50.0
Advanced Degree	18	25.0
Parent Occupation		
Employed for wages	63	87.5
Self-employed	6	8.3
Out of work and looking	1	1.4
Out of work and not looking	1	1.4
A homemaker	0	0.0
A student	0	0.0
Military	0	0.0
Retired	0	0.0
Unable to work	1	1.4
Marital Status		
Married	60	83.3
Single	4	5.6
Separated	3	4.2
Divorced	3	4.2
Widowed	2	2.8
Living with someone	0	0.0
Age of Child (Years)		
6–8	44	61.1
9–11	12	16.7
12–14	10	13.9
15–18	6	8.4
Age of Diagnosis (Years)		
0–2	26	36.1
3–5	39	54.2
6–8	7	9.7
Gender of Child		
Male	58	80.6
Female	14	19.4
Diagnostic Severity		
Mild	35	48.6
Moderate	28	38.9
Severe	9	12.5

**Table 2:** School Placement Based on Diagnostic Severity Level.

Variable	*n*	*%*
Mild		
Public School	26	36.1
Special Needs School	3	4.2
Home Program	3	4.2
Residential	0	0
Medical Facility	3	4.2
Moderate		
Public School	18	25.0
Special Needs School	4	5.5
Home Program	1	1.4
Residential	2	2.7
Medical Facility	3	4.2
Severe		
Public School	5	6.9
Special Needs School	3	4.2
Home Program	0	0
Residential	1	1.4
Medical Facility	0	0

Note. Percentages are out of complete sample.

**Table 3:** Descriptive Statistics for Predictor and Outcome Variable.

Variable	*n*	*M*	*SD*	Min.	Max.
Autism Index	72	86.4	18.9	53.0	125.0
Adaptive Skills	72	83.6	22.5	41.0	120.0
School Placement	72	1.7	1.3	1.0	5.0
Hours of ABA	72	12.5	9.5	2.0	42.0
Length of Time	72	15.8	11.9	1.0	48.0

Note. Autism Index = points obtained on GARS-3; ABA = applied behavioral analysis.

**Table 4:** Parent Report of Early Intervention Variables: Correlations.

Variables	1	2	3	4	5	6	7	8	9	10	11	12	13	14	15	16	17
1. Age	1.00																
2. Gender	0.13	1.00															
3. AD	−0.19	0.06	1.00														
4. SEVD	0.45 **	0.00	−0.14	1.00													
5. PMS	0.23 *	0.05	−0.12	0.31 **	1.00												
6. PO	0.01	−0.10	0.16	0.31 **	0.15	1.00											
7. PED	−0.05	0.07	−0.13	0.05	−0.04	−0.24 *	1.00										
8. AABA	−0.22	0.10	0.55 **	−0.31 **	−0.10	0.05	−0.17	1.00									
9. LEI	0.18	−0.11	−0.39 *	−0.09	0.07	0.03	0.21	−0.45 **	1.00								
10. HABA	0.03	−0.23	−0.06	0.01	−0.19	−0.05	−0.18	−0.03	0.12	1.00							
11. EISET	−0.16	−0.19	−0.19	0.14	0.07	−0.17	0.18	−0.26	0.12	−0.07	1.00						
12. SCSET	0.14	0.14	0.02	0.05	0.05	−0.06	−0.06	0.09	−0.29 *	−0.21	0.12	1.00					
13. EABA	0.02	0.01	−0.09	0.24 *	−0.00	−0.14	−0.02	−0.28 *	0.20	0.08	0.26 *	−0.14	1.00				
14. OQOL	−0.07	−0.24 *	−0.06	0.28 *	0.01	−0.01	0.02	−0.13	0.17	0.15	0.25 *	−0.12	0.49 **	1.00			
15. AI	0.21	−0.13	−0.14	0.29 *	−0.03	0.16	0.00	−0.19	−0.01	−0.04	−0.08	0.01	0.05	0.14	1.00		
16. GSEV	0.14	−0.19	−0.05	0.32 **	0.01	0.20	0.04	−0.17	−0.05	−0.03	−0.10	−0.01	0.05	0.20	0.91 **	1.00	
17. GAC	−0.46 **	0.10	0.44**	−0.42 **	−0.10	−0.16	−0.10	0.54 **	−0.46 **	0.02	−0.36 **	0.12	−0.20	−0.11	−0.29 *	−0.22	1.00

Note. Age = current age, AD = age at diagnosis, SEVD = diagnostic severity, PMS = parent marital status, PO = parent occupation, PED = parental education, AABA = age began ABA intervention, LEI = length of ABA intervention, HABA = hours per week of ABA, EISET = early intervention setting, SCSET = current school setting, EABA = effectiveness of ABA, OQOL = child’s overall quality of life, AI = GARS Autism Index, GSEV = GARS Autism Severity, GAC = ABAS-3 General Adaptive Composite. * *p* < 0.05. ** *p* < 0.01.

**Table 5:** Hierarchical Regression Analysis for Prediction of Autism Symptom Severity.

	Model 1			Model 2		
Variable	B	SE	β	B	SE	β
Age	0.716	0.731	0.127	0.830	0.751	0.147
Gender	−6.834	5.484	−0.144	−8.175	5.727	−0.172
Severity	6.440	3.481	0.237	6.399	3.511	0.236
Hours				−0.151	0.237	−0.076
Length				−0.118	0.190	−0.074
*R* ^2^	0.115			0.127		
Adjusted *R*^2^	0.076			0.061		
*F* Change	2.952			0.436		

Note. *df* for Model 1 = 68; *df* for Model 2 = 66.

**Table 6:** Hierarchical Regression Analysis for Prediction of Adaptive Functioning.

	Model 1			Model 2		
Variable	B	SE	β	B	SE	β
Age	−2.440	0.781	−0.363 **	−1.966	0.721	−0.293 **
Gender	8.563	5.854	0.151	6.942	5.504	0.123
Severity	−8.155	3.715	−0.253 *	−8.044	3.374	−0.249 *
Hours				0.245	0.228	0.103
Length				−0.729	0.182	−0.384 **
*R* ^2^	0.287			0.430		
Adjusted *R*^2^	0.255			0.386		
*F* Change	9.117			8.259		

Note. *df* for Model 1 = 68; *df* for Model 2 = 66; * *p* < 0.05; ** *p* < 0.01.

**Table 7:** Hierarchical Regression Analysis for Prediction of School Placement.

	Model 1			Model 2		
Variable	B	SE	β	B	SE	β
Age	0.049	0.051	0.130	0.076	0.049	0.203
Gender	0.396	0.381	0.125	0.135	0.374	0.043
Severity	−0.009	0.242	−0.005	−0.014	0.229	−0.008
Hours				−0.023	0.015	−0.170
Length				−0.032	0.0123	−0.303 *
*R* ^2^	0.036			0.159		
Adjusted *R*^2^	−0.006			0.096		
*F* Change	0.858			4.823		

Note. *df* for Model 1 = 68; *df* for Model 2 = 66. *p* < 0.10. * *p* < 0.05.

**Table 8:** Comparison and Integration of Findings from Quantitative Analyses with the Comparable Qualitative Findings.

Variable	Quantitative Results	Qualitative Results	Convergence or Divergence of Data
Autism Symptom Severity	The intensity of ABA is not a unique predictor.	Parents reported ABA to be a useful tool for their child’s behaviors (Theme 1).	Divergence
Adaptive Skills	The intensity of ABA is a unique predictor and accounts for 14.3% of the variance.	Parents reported ABA to be a useful tool to increase adaptive skills (Theme 2).	Convergence
School Placement	The intensity of ABA is a unique predictor and accounts for 12.3% of the variance.	Parents reported ABA to be a useful tool before school began (Theme 1).	Convergence

## Data Availability

Data is not publicly available due to privacy and ethical restrictions specified in our IRB document. However, if researchers are interested in obtaining the related data, they could reach out the corresponding author at yding4@fordham for individual use.
